# Structural and Functional Insights into *Saccharomyces cerevisiae* Riboflavin Biosynthesis Reductase RIB7

**DOI:** 10.1371/journal.pone.0061249

**Published:** 2013-04-19

**Authors:** Zongyang Lv, Jian Sun, Yingfang Liu

**Affiliations:** 1 National Laboratory of Biomacromolecules, Institute of Biophysics, Chinese Academy of Sciences, Beijing, China; 2 University of the Chinese Academy of Sciences, Beijing, China; University of South Florida College of Medicine, United States of America

## Abstract

*Saccharomyces cerevisiae* RIB7 (ScRIB7) is a potent target for anti-fungal agents because of its involvement in the riboflavin biosynthesis pathway as a NADPH-dependent reductase. However, the catalytic mechanism of riboflavin biosynthesis reductase (RBSRs) is controversial, and enzyme structure information is still lacking in eukaryotes. Here we report the crystal structure of *Saccharomyces cerevisiae* RIB7 at 2.10 Å resolution and its complex with NADPH at 2.35 Å resolution. ScRIB7 exists as a stable homodimer, and each subunit consists of nine central β-sheets flanked by five helices, resembling the structure of RIB7 homologues. A conserved G^76^-X-G^78^-X_n_-G^181^-G^182^ motif is present at the NADPH pyrophosphate group binding site. Activity assays confirmed the necessity of Thr79, Asp83, Glu180 and Gly182 for the activity of ScRIB7. Substrate preference of ScRIB7 was altered by mutating one residue (Thr35) to a Lysine, implying that ScRIB7 Thr35 and its corresponding residue, a lysine in bacteria, are important in substrate-specific recognition.

## Introduction

Riboflavin is the precursor of FAD and FMN, ubiquitously used as coenzymes in redox and non-redox processes [1]. Riboflavin plays a role in the development and maintenance of the surface structures of corneal epithelial cells, regulating the structure and function of the ocular surface [2]. Human liver cells deficient in riboflavin experience ER stress and decreased apolipoprotein B-100 secretion [3]. Deficiencies in riboflavin biosynthesis are reported to affect tetrapyrrole biosynthesis in plant cells [4]. While humans must obtain this vitamin from dietary sources, riboflavin is biosynthesized in plants and many microorganisms. Some of the microorganisms lack the riboflavin salvage pathway and are unable to absorb riboflavin from the environment. Riboflavin biosynthetic enzymes and enzymes of the riboflavin salvage pathway thus have potential as novel candidate antibiotic drug targets, especially for treating drug resistant microbes [5], [6]. In addition, structures of riboflavin biosynthesis reductases were shown to be very similar to dihydrofolate reductases [7], [8], [9], and inhibitors of dihydrofolate reductases such as methotrexate, pyrimethamine, and trimethoprim, have long been used clinically in the treatment of cancer and bacterial and fungal infections [10].

Most of the catalytic enzymes involved in riboflavin biosynthetic processes have been characterized, and the riboflavin biosynthesis pathway has been reviewed in detail [11]. Riboflavin biosynthesis in different organisms share similar convergent pathways whose major differences occur in the two reaction steps where intermediate 2,5-diamino-6-ribosylamino-4(3H)-pyrimidinone 5′-phosphate (DAROPP) is finally converted to intermediate ARIPP [11]. In archaea and fungi, DAROPP is reduced at the phosphoribosyl moiety by RIB7 yielding 2, 5-diamino-6-ribitylamino-4(3H)-pyrimidinone 5′-phosphate (DARIPP), which is then deaminated into 5-amino-6-ribitylamino-2,4 (1H,3H)-pyrimidinedione 5′-phosphate (ARIPP) by RIB2 [7], [11], [12], [13]. In most eubacteria, a bifunctional enzyme RibD first catalyzes deamination then reduction reactions [8], [9], [11]. The deaminase domain of RibD is usually located at the N-terminal of the enzyme and catalyzes the formation of 5-amino-6-ribosylamino-2,4(1H,3H)-pyrimidinedione 5′-phosphate (AROPP) while the reductase domain is usually located at the C-terminal and catalyzes the formation of ARIPP [8], [9], [11], [14]. Sequence homology between fungal and archaeal RIB7 and the eubacterial RibD reductase domain is lower compared to enzymes catalyzing other steps in riboflavin biosynthesis [11].

The underlying mechanism of riboflavin biosynthesis type reductases has been studied both by structural and functional analyses. Isotope feeding experiments in *Ashbya gossypii* ruled out an Amadori rearrangement for the reduction mechanism, and pointed to a Schiff base pathway [15]. Several RBSR structures have been determined (Table 1). The structure of *Escherichia coli* RibD (EcRibD) in a binary complex with substrate analog ribose-5-phosphate or oxidized cofactor NADP+, and sequence alignment information indicate that the conserved residue Asp200 is a catalytic residue [8]. *Bacillus subtilis* RibG (BsRibG) in complex with intermediate AROPP has led to the proposal of a different catalytic mechanism: Glu290 rather than the previously proposed aspartate (Asp200 in EcRibD) initiates the proton transfer process [9]. Activity of RBSR mutants of the proposed residues are not measured, and the specific residue involved in catalysis is still controversial. EcRibD Lys152 and BsRibG Lys151 are both thought to determine substrate-specific recognition in bacteria RBSRs [8], [9]. However, little experimental evidence has been provided and information on eukaryotic riboflavin biosynthesis reductase is also lacking.

**Table 1:** Available structures of RBSRs.

Species	Protein name	PDB code	Reference
*Methanocaldococcus jannaschii*	RIB7	2AZN	J. Mol. Biol. (2006) 359, 1334–1351
*Bacillus subtilis*	RIBG	2B3Z, 2D5N, 3EX8	J. Biol. Chem. (2006) 281, 7605–7613, J. Biol. Chem. (2009) 284, 1725–1731
*Escherichia coli*	RIBD	2G6V, 2OBC, 2O7P	J. Mol. Biol. (2007) 373, 48–64
*Thermotoga maritime*	TM1828	2HXV	Unpublished (Joint Center for Structural Genomics)

In this study we have solved the structure of *Saccharomyces cerevisiae* RIB7, providing the first eukaryotic riboflavin biosynthesis reductase structure, and its binary structure with NADPH. A G^76^-X-G^78^-X_n_-G^181^-G^182^ motif is found at the NADPH pyrophosphate group binding site. Several ScRIB7 mutants were constructed based on structural information and their reductase activity was examined. Results showed both mutation in Asp83 and Glu180 lowered the activity of the enzyme. We have also changed the substrate preference by mutating one residue (Thr35) to a Lysine.

## Experimental Procedures

### Cloning of *Escherichia Coli RibD, Saccharomyces* *Cerevisiae RIB1* and *RIB7*

Synthetic oligonucleotide primers (sequences provided in Table S1), genomic DNA and standard PCR conditions were used to amplify the *RibD* gene from *Escherichia coli* and the *RIB1* and *RIB7* genes from *Saccharomyces cerevisiae* and introduce unique restriction enzyme sites: *BglII*/*XhoI* for *RIB1* and *BamHI*/*XhoI* for *RIB7* [16]. The PCR products were digested and gene products were ligated into pGEX6p-1/pRSFduet-1 expression vectors, previously digested with the *BamHI* and *XhoI* enzymes. The recombinant plasmids pGEX6p-1-*RIB7*, pRSFduet-1-*RibD*, and pRSFduet-1-*RIB1* were sequenced to confirm their identity.

### Site-directed Mutagenesis

Mutations were introduced into plasmid pGEX6p-1-*RIB7* by site-directed mutagenesis PCR method using a high-fidelity polymerase Superstar from Genestar [17]. Primer information can be found in the Supplementary Information.

PCR products were digested with *Dpn*1, and transformed into *E. coli* DH5α competent cells. Clones were sequenced to verify that the selected clones contain the desired mutations.

### Protein Preparation

*E. coli* BL21 (DE3) cells were transformed respectively with recombinant plasmids pGEX6p-1-*RIB7*, pRSFduet-1-*RibD* and pRSFduet-1-*RIB1*, then inoculated into LB medium containing ampicillin or kanamycin antibiotics, and induced at 37°C with 0.1 mM IPTG for 5 h.

*E. coli* cells expressing GST-RIB7 (∼6 l LB) were harvested and resuspended in 300 ml GST buffer (PBS, 1 M NaCl), and lysed by French press. After removal of cellular debris by centrifugation at 30,000 g at 4°C for 30 min, the supernatant was applied to Glutathione-Sepharose beads (∼20 ml, GE Healthcare). The beads were collected and washed with excess GST buffer. PreScission protease was added to remove the GST tag. After 16 h at 4°C, the eluate was collected and concentrated to ∼1 ml then loaded onto Superdex 200 column (Amersham Biosciences) equilibrated with 20 mM Tris-HCl (pH 8.0), 150 mM NaCl. Peaks were analyzed by SDS-PAGE and the corresponding fractions were pooled and concentrated to ∼10 mg/ml for crystallization. ScRIB7 mutants for activity assays were prepared as above except they were not submitted to gel filtration. Mutants were concentrated to ∼2 mg/ml.

*E. coli* cells expressing 6*his-RibD or 6*his-RIB1 (∼2 l LB) were harvested and resuspended with 100 ml buffer A containing 10 mM imidazole, 50 mM Tris-HCl (pH 8.0), 500 mM NaCl. Cells underwent similar procedures to those for ScRIB7, including lysis by French Press and centrifugation. The clear soluble fraction was loaded onto an IDA nickel column pre-equilibrated with buffer A, and then washed with 200 ml buffer B containing 50 mM imidazole, 50 mM Tris-HCl (pH 8.0), 500 mM NaCl. Proteins were eluted with 20 ml buffer C containing 500 mM imidazole, 50 mM Tris-HCl (pH 8.0), 500 mM NaCl, pooled, and concentrated to a final concentration of ∼2 mg/ml. Enzymes were fractioned and stored at −80°C for subsequent experiments.

Seleno-derivative ScRIB7 was expressed using M9 minimal medium lacking methionine and supplemented with 25 mg/l seleno-methionine, and purified according to the same procedures used for the native protein.

### Protein Crystallization

Native and seleno-derivative ScRIB7 crystals were grown using the hanging drop vapour diffusion method. An equal volume (2 µl) of protein solution (10 mg/ml) was mixed with reservoir solution. The crystals appeared under a 0.05 M calcium chloride dihydrate, 0.1 M BIS-TRIS (pH 6.5), 30% v/v Polyethylene glycol monomethyl ether 550 condition. Crystals were directly flash frozen in liquid nitrogen.

Seleno-derivative and native crystal X-ray diffraction data were collected at beamlines I04 at the Diamond Synchrotron Facility (London, UK) and BL17U at the Shanghai Synchrotron Radiation Facility (Shanghai, China), respectively. The crystal belongs to the P2_1_2_1_2_1_ space group and has two molecules in each asymmetric unit. A NADPH derivative was prepared by soaking crystals in reservoir solution containing 10 mM NADPH for two weeks. X-ray diffraction data were collected at beamline MX1 at the Australia Synchrotron Facility (Melbourne, Australia). Diffraction data sets were processed with HKL2000 [18].

### Structure Determination

The ScRIB7 crystal structure was determined using single-wavelength anomalous dispersion (SAD) of the selenium-substitution derivative. Eight Selenium positions were identified with SHELXD [19]. The initial phase was calculated by Phaser and improved by DM [20], [21], [22]. Some β-sheets were observed but were hard to model because of the low resolution of the Seleno-derivative data. Phase extension was used to combine low-resolution Seleno-derivative phase information and high-resolution native amplitude information to produce a better electron density map. Buccaneer and Phenix.autobuild were used in automated model building [23], [24]. The structure was completed by manual building in Coot [25]. The NADPH derivative structure was resolved by molecular replacement with Phaser using a ligand-free model [22]. The statistics for data collection and refinement are summarized in Table 2. In the ligand-free model, 94.69% of the residues were in the most favored region of the Ramachandran plot, 4.83% were in the additional allowed region and 0.48% were in the disallowed region. In the ScRIB7 NADPH binary complex model, about 94.25% of the residues were in the most favored region of the Ramachandran plot, 5.5% were in the additional allowed region and 0.25% were in the disallowed region (Table 2) [26]. All figures were prepared with PyMol [27]. The atomic coordinates and structure factors (codes 4HA7 and 4HA9) have been deposited in the Protein Data Bank, Research Collaboratory for Structural Bioinformatics, Rutgers University, New Brunswick, NJ (http://www.rcsb.org).

**Table 2:** Statistics for data collection and structural refinement.

Data collection statistics			
Crystal	Seleno-derivative	Native	NADPH complex
Beamline	Diamond I04	SSRF BL17U	Australian synchrotron MX1
Wavelength (Å)	0.9796	0.9793	0.9184
Space group	P2_1_2_1_2_1_	P2_1_2_1_2_1_	P2_1_2_1_2_1_
Unit Cell (Å)	44.9, 68.1, 144.0	45.5, 68.6, 149.0	46.0, 68.9, 150.3
Resolution (Å)	3.60	2.10	2.35
Rsym (%)^a^	13.7 (66.3)^b^	9.7 (67.9)	5.6 (59.1)
<I/σ>	38.4(5.3)	17.8(4.1)	63.5(6.6)
Redundancy	13.6 (14.4)	6.9 (7.3)	12.6 (12.5)
Unique reflection	5499	27560	20487
Completeness (%)	100 (100)	98.5 (100)	99.8 (100)
**Refinement statistics**			
Resolution (Å)		36.75-2.10	34.43-2.35
Reflection used for refinement		26185	19437
Reflection used for test set		1375	1050
Final R/R_free_ c (%)		20.00/26.60	20.51/26.49
r.m.s.d. dbonds/angles		0.008 Å/1.235°	0.008 Å/1.309°
Mean B value (Å^2^)		59.9	55.7
Protein/solvent atoms		3373/141	3348/70
NADPH atoms			48
NADPH mean B factor (Å^2^)			84.6
Ramachandran plot			
Most favorable		94.69%	94.25%
Additionally allowed		4.83%	5.5%
Disallowed		0.48%	0.25%
**PDB entry**		4HA7	4HA9

a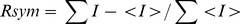, where I is the intensity and <I> is the averaged intensity of multiple measurements. The summation is for all measured reflections.

b Values in parentheses are those of the highest resolution shell.

c Calculated from about 5% of the reflections set aside during refinement.

d r.m.s.d., root mean square deviation.

### Analytical Ultracentrifugation

The molecular mass of wild type ScRIB7 in solution was estimated with a Beckman-Coulter ProteomeLab XL-I analytical ultracentrifuge using a 60Ti rotor. Sedimentation velocity experiments were performed at 20°C and 60,000 rpm with standard double sector centerpieces. Protein sample was prepared as OD_280_ absorbance about 0.8 in buffer containing 20 mM Tris-HCl (pH 8.0) and 150 mM NaCl. The UV absorption of the cells was scanned every 30 sec for 4 h, and data were analyzed using Sedfit [28].

### Enzymatic Synthesis of DAROPP and AROPP

Enzymatic synthesis of the substrates and the reductase assays were modified from the reported methods [8], [9]. The reaction mixture for the synthesis of DAROPP contained 50 mM Tris-HCl (pH 7.5), 8 mM MgCl_2_, 5 mM GTP, 100 mM NaCl, 20 mM DTT and 2 mg/ml ScRIB1 [29]. After incubation at 25°C, the generation of DAROPP was monitored with absorbance at OD_292 nm_. The enzyme was removed by boiling the mixture at 95°C for 5 min and centrifugation for 20 min at 20,000 g. For generation of AROPP, excess EcRibD (10 mg/ml) was added to the DAROPP solution and incubated at 37°C for 30 min. EcRibD was also removed by boiling and centrifugation.

### Reductase Activity Assay

To measure the relative reductase activity of the various ScRIB7 mutants, about 10 µM reductase and 1 mM NADPH were added to the DAROPP or AROPP solutions and incubated for 60 min at 37°C. Diacetyl was added to a final concentration of 1% (v/v). The fluorescence spectrum was recorded at an excitation wavelength of 415 nm. The relative reductase activity was estimated in three independent experiments by subtracting the protein background emission from the emission intensity at 482 nm. Data were analyzed using Origin (OriginLab, Northampton, MA).

### ITC Assay

Interactions between ScRIB7, various mutants and NADPH were detected by isothermal titration calorimetry using nano-ITC2g. The protein was dialyzed into PBS and diluted to 80 µM. It was then titrated by 800 µM NADPH dissolved in the same buffer (10 µl NADPH was injected every 300 s). The interaction heat flow was monitored to calculate their affinity. Results were analyzed with NanoAnalyze (TA Instruments).

## Results and Discussion

### Overall Structure

**Figure 1:**
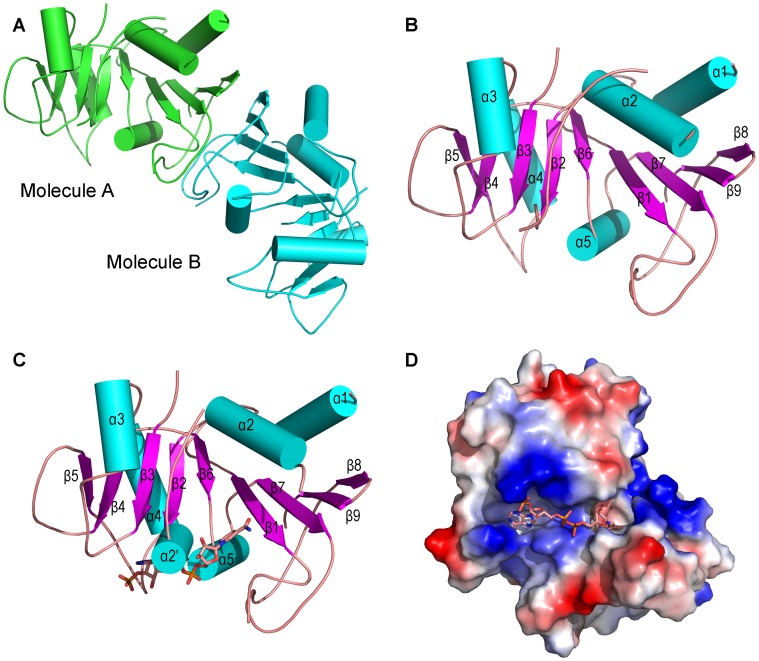
Overall structure of ScRIB7 and its NADPH binary complex. A, ScRIB7 homodimer. The dimer is viewed along the noncrystallographic 2-fold axis. Molecules A and B are colored in green and cyan, respectively. B, ScRIB7 monomer. Secondary structures are numbered according to the primary sequence. C, ScRIB7-NADPH binary complex. An additional helix α2′ is formed upon NADPH cofactor binding. D, Surface electrostatic distribution of the ScRIB7-NADPH binary complex. Positively charged residues are in blue, negatively charged residues are in red and neutral residues are in white. NADPH is shown as sticks: carbon (pink), oxygen (red), nitrogen (blue).

**Figure 2:**
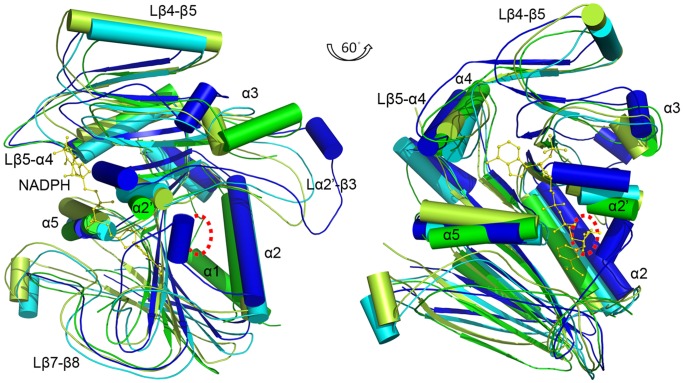
The structure superposition of ScRIB7-NADPH binary complex with homolog structures. ScRIB7-NADPH chain A is superposed with the cofactor binding structures of MjRIB7 (2AZN, chain A), EcRibD reductase domain (2O7P, chain A) and BsRibG reductase domain (2D5N, chain B). ScRIB7 is shown in green, NADPH bound to ScRIB7 is shown as yellow sticks, MjRIB7 is shown in limon, EcRibD is shown in blue, BsRibG is shown in cyan. The overall structure of ScRIB7 and available homolog structures are very similar, still some significant differences are present. ScRIB7 has a unique α1 compared to MjRIB7, while EcRibD and BsRibG have N-terminal deaminase domains (not shown). Lβ4–β5 is similar to the loop in EcRibD; while the corresponding residues formed α-helices in MjRIB7 and BsRibG (Lβ4–β5 is invisible for it locates inside the helices of MjRIB7 and BsRibG). Lβ5-α4 is longer than those in available homolog structures while Lβ7–β8 is shorter. The substrate binding site between α2 and α2′ is schematically shown as a red dotted circle.

The structure of ScRIB7 was solved using single-wavelength anomalous dispersion (SAD). The crystals belong to space group P2_1_2_1_2_1_, with one asymmetric unit containing two ScRIB7 molecules (Table 2 & Fig. 1). The RMSD between the C-alpha atoms of the two subunits is 0.3 Å. Each monomer is a mixed α/β structure: nine central β-strands flanked by five helices which are similar to those of the reductase domain of known RBSRs (Table 1 & Fig. 2). Secondary structural elements were numbered according to the primary sequence: β1–β9, α1–α5 (Fig. 1B).

**Figure 3:**
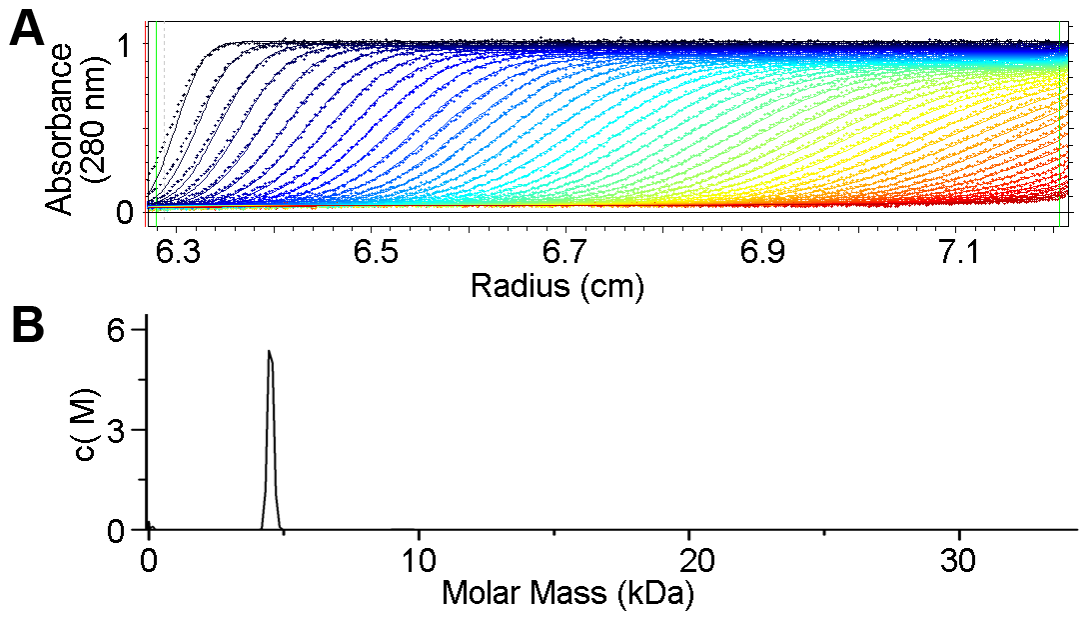
Sedimentation velocity analysis of ScRIB7. A, The raw sedimentation signals acquired at different time points. Protein sample was prepared as OD_280_ absorbance about 0.8 in buffer containing 20 mM Tris-HCl (pH 8.0) and 150 mM NaCl. The sample was scanned at intervals of 30 s for 4 h. (b) Continuous molar mass distribution of the protein showing a single peak with a molecular mass of ∼43 kDa, which is closest to the mass of a ScRIB7 dimer.

Previously reported riboflavin biosynthesis reductases have different oligomer states. MjRIB7 and EcRibD were dimeric, while BsRibG was a tetramer in solution [7], [8], [9]. Here, gel-filtration experiments indicated that ScRIB7 was in a dimeric state (data not shown). Further sedimentation analysis verified this result: the enzyme sedimented as a dimer (Fig. 3). The most likely dimeric interface was calculated using PISA, and the two molecules per asymmetric unit were modeled in the appropriate positions calculated by PISA [30]. The size of the calculated interaction area was 1,303 Å^2^. The two subunit interfaces are made up of the four β-sheets (β1, β7–β9) and several loops (Lβ7–β8, Lβ8–β9). There are nineteen hydrogen bonds, and seven salt bridges between the protein atoms across the interface and the extensive hydrophobic surface formed by residues Tyr36, Leu40, Ala42, Leu190, Leu199, Ile201, Ile203, Phe207, Leu208, Val215, Val221, Leu223, Met226, Trp228, Trp229, Ile232, Val235, and Leu237.

The overall structure of ScRIB7 and available homolog structures (Table 1) are very similar that the calculated RMSD values are: 2.0–2.3 Å, still some significant differences are present. ScRIB7 has a unique α1 compared to MjRIB7, while EcRibD and BsRibG have N-terminal deaminase domain (Fig. 2). Lβ4–β5 is similar to the loop in EcRibD, while corresponding residues formed α-helices in MjRIB7 and BsRibG (Fig. 2). Loop Lβ5-α4 is longer than those in available homolog structures while loop Lβ7–β8 is shorter (Fig. 2). Electronic density of loops Lα2′-β3 and Lβ1–α2 in ScRIB7 are missing probably due to their flexibility.

### ScRIB7-NADPH Binding Site

Crystals of ScRIB7 in binary complex with NADPH were obtained by adding the cofactor NADPH to the original crystal growing solution. NADPH was modeled into the clear electron density observed on one molecule of the ScRIB7 dimer (Fig. S1). The enzyme structure did not show significant change upon cofactor binding (RMSD between C-alpha atoms was 0.26 Å) except that an additional helix α2′ was formed by residues 79–82.

**Figure 4:**
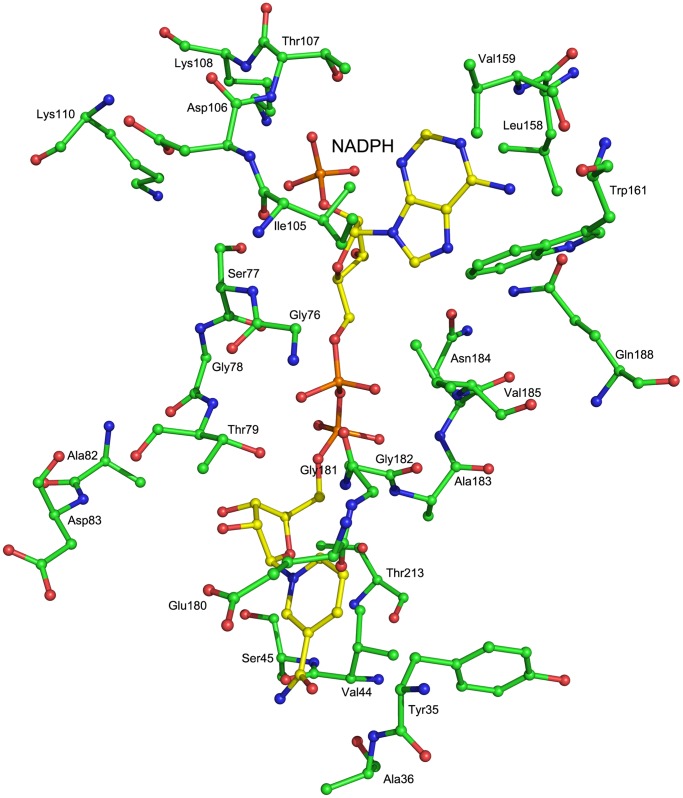
The cofactor binding site of ScRIB7 chain A. Residues of ScRIB7 that are within 4 Å around the cofactor and Asp83 are shown. Both ScRIB7 and the cofactor NADPH are shown as sticks: carbon from ScRIB7 (green), carbon from NADPH (yellow), oxygen (red), and nitrogen (blue).

The overall NADPH binding architecture is very similar to that of known RBSR structures, but has some minor differences. The adenosine 2′-phophate moiety binding pocket is formed by Thr107, Lys108, Leu158, Val159, Trp161, Asn184, Val185, Gln188, and Leu189 (Fig. 4). Lys108 occupies the same position as that of Lys84 in MjRib7 that favors the binding of the phosphate group of NADPH; however, residues carrying different charges are present in the EcRibD and BsRibG structures (EcRibD Gln235 and BsRibG Val222). ScRIB7 Trp161 has a much larger side chain than the equivalent residues in its homologs, and provides a much larger hydrophobic stereo blockade.

**Figure 5:**
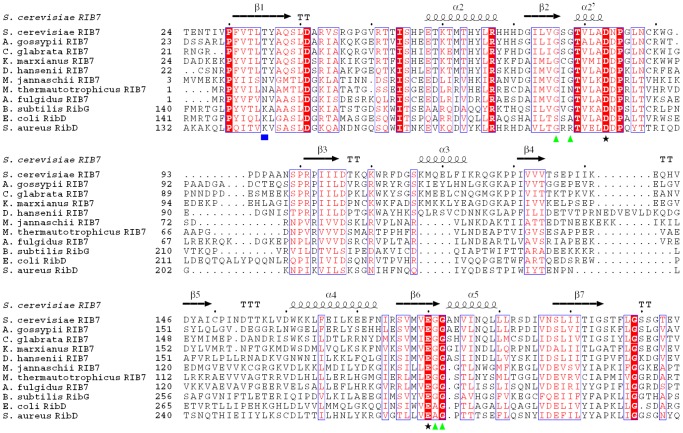
Multiple-sequence alignment of ScRIB7 with RBSRs from different organisms. The multisequence alignment was performed using ClustalW2 and ESPript [35], [38]. Secondary structural elements of ScRIB7 are displayed above the sequence. All sequences were downloaded from the ExPASy database (UniProt accession numbers in parentheses): *S. cerevisiae* RIB7 (P33312), *A. gossypii* RIB7 (Q757H6), *C. glabrata* RIB7 (Q6FU96), *K. marxianus* RIB7 (Q9P4B8), *D. hansenii* RIB7 (Q6BII9), *M. jannaschii* Rib7 (Q58085), *M. thermautotrophicus* RIB7 (O26337), *A. fulgidus* RIB7 (O28272), *B. subtilis* RibG (P17618), *E. coli* RibD (P25539), *S. aureus* RibD (D0K610). Filled blue rectangle: key residues involved in substrate-specific recognition; filled black stars: key residues that may play key roles in reduction catalysis; filled green triangle: glycines of G^76^-X-G^78^-X_n_-G^181^-G^182^ motif. An additional helix α2′ is formed upon NADPH cofactor binding. Sequences are not shown in integrity.

**Figure 6:**
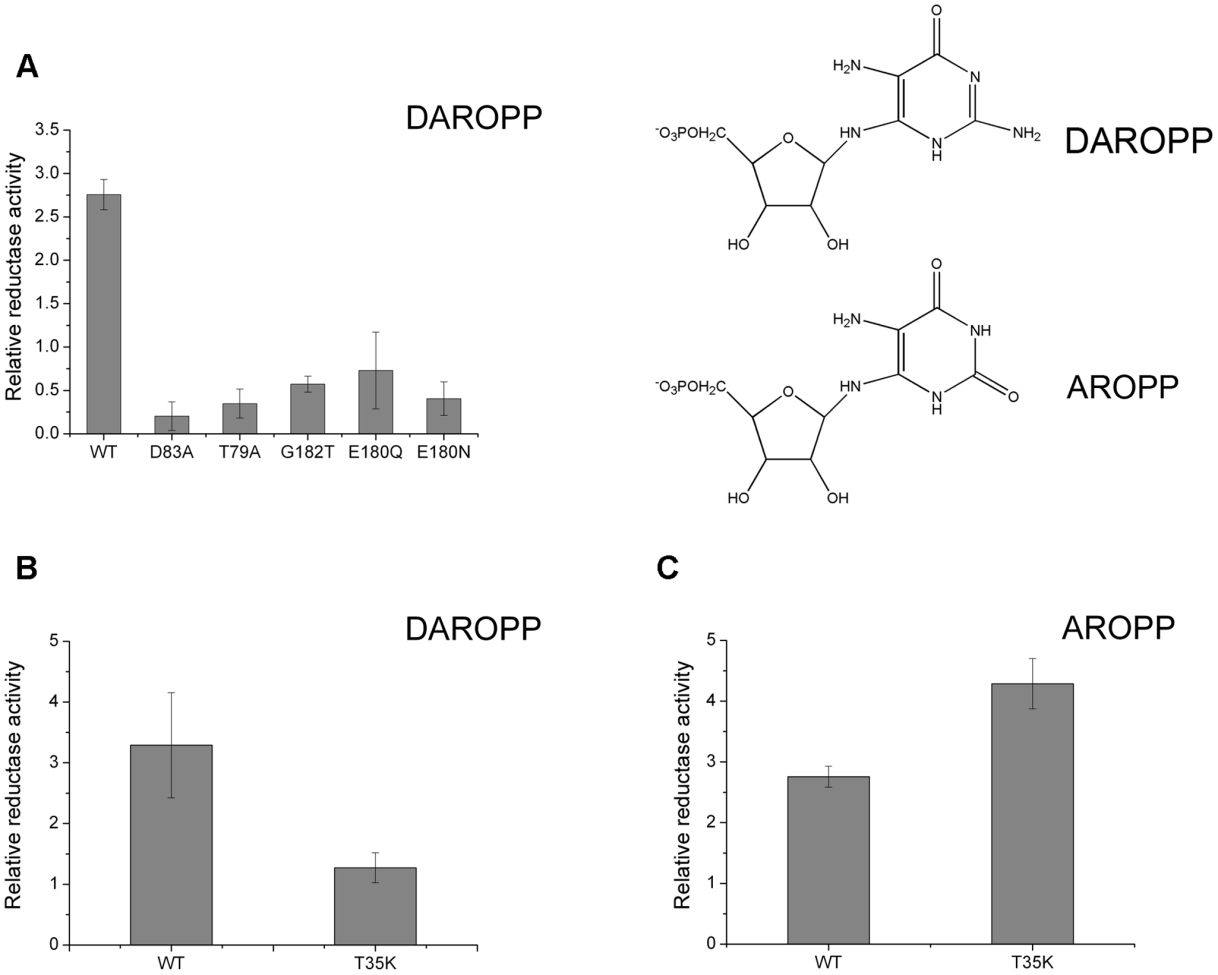
Enzyme activity assays of ScRIB7 mutants. A, Relative reductase activity of ScRIB7 mutants toward DAROPP. B&C, Relative reductive activity of T35K mutant and wild type ScRIB7 toward DAROPP or AROPP as substrates. DAROPP and AROPP molecular structures are shown in the inset. Results are presented as means ± S.D. of three independent experiments.

The turn between β2 and α2′ and the loop between β6 and α5 form a narrow channel that accommodates the pyrophosphate group of NADPH. The residues that make up this channel are rich in glycine. Like the G-X-X-G-X-X-G fingerprint or glycine rich motifs that are prevalent in reductases, in ScRIB7, a G^76^-X-G^78^ fingerprint and two glycine residues G^181^-G^182^ that are in close proximity to the cofactor form a G^76^-X-G^78^-X_n_-G^181^-G^182^ motif (Fig. 4) [31], [32], [33]. Sequence alignment showed these glycine residues are conserved among fungi and most archaea (Fig. 5). Larger side chains at this channel might prevent cofactor binding. Thr79 occupies a critical position that is adjacent to both the nicotine and pyrophosphate portions of NADPH (Fig. 4). The distance between its hydroxyl oxygen and a pyrophosphate oxygen is 2.76 Å. A hydrogen bond is formed between them. Mutation of Thr79 to a hydrophobic residue may broke the hydrogen bond with the cofactor and reduce NADPH binding. As predicted, T79A and G182T mutants displayed loss in enzyme activity, which is consistent with their proposed roles, probably due to the reduced ability in NADPH binding (Fig. 6A).

### Substrate Binding Site and Recognition

The substrates of archaea and fungal RIB7 (DAROPP) and plant and bacterial RibD/G (AROPP) are very similar except for the C2 position of the pyrimidine ring: DAROPP contains an amine group while AROPP has a double bonded oxygen [11] (Fig. 6 inset). When RibD/G reductases are simultaneously exposed to DAROPP and AROPP, they are able to specifically recognize AROPP as their substrate [8], [9], [34]. Their structures show that the reductase catalytic site of bacterial RibD/G is some distance from the deaminase catalytic site, so it seems unlikely that substrates are transported within the enzyme to ensure the correct reaction sequence [8], [9]. A lysine residue (EcRibD Lys152/BsRibG Lys151) seems to form favourable interactions with the oxygen at the C2 position of the pyrimidine ring in AROPP but not with the amino group at the same position in DAROPP [8], [9]. This residue has thus been proposed to account for this substrate-specific recognition, but no experimental evidence has been reported.

Since Thr35 in ScRIB7 is at the site corresponding to EcRibD Lys152/BsRibG Lys151, we generated a T35K mutant to test its activity towards AROPP and DAROPP. AROPP is not commercially available, so it was prepared as described before [9]. Results showed that AROPP was recognized much better (by about 3 fold) by the T35K mutant than DAROPP, while the wild type was better at recognizing DAROPP (Fig. 6B). The side chain of threonine is smaller than that of lysine, so the amine group, larger than an oxygen molecule, can be accommodated. The apical amine of Lys35 in mutant T35K repels the amine of DAROPP but forms favorable interactions with oxygen. Therefore, ScRIB7 Thr35 favors DAROPP binding, though ScRIB7 is not exposed to AROPP *in vivo*; EcRibD Lys152 and BsRibG Lys151 are important residues for specific AROPP recognition in bacteria. Our conclusion is consistent with previously proposed ideas and the sequence alignment demonstrates that the threonine in fungi and the lysine in eubacteria are conserved, while archaea encodes asparagines at the same place (Fig. 5).

### Exploration of the Reduction Mechanism

Although RBSRs are not very conserved compared to other enzymes in the riboflavin biosynthesis process, a number of invariant residues are observed, and the available structures of RBSRs are very similar (Fig. 2 & Fig. 5) [7], [8], [9], [35], [36]. RBSRs probably utilize the same core reduction mechanism: a conserved ionizable residue was proposed to initiate the reaction by abstracting a proton from the substrate, however, two different residues were proposed as mentioned above: Asp83 and Glu180 (ScRIB7 numbering) [7], [8], [9]. They are the only two ionizable residues at the reaction center, and their corresponding residues in BsRibG have similar distance to the substrate in the complex structure [9]. Further evidences are needed to provide a more reasonable explanation for the reduction mechanism of RBSRs.

**Figure 7:**
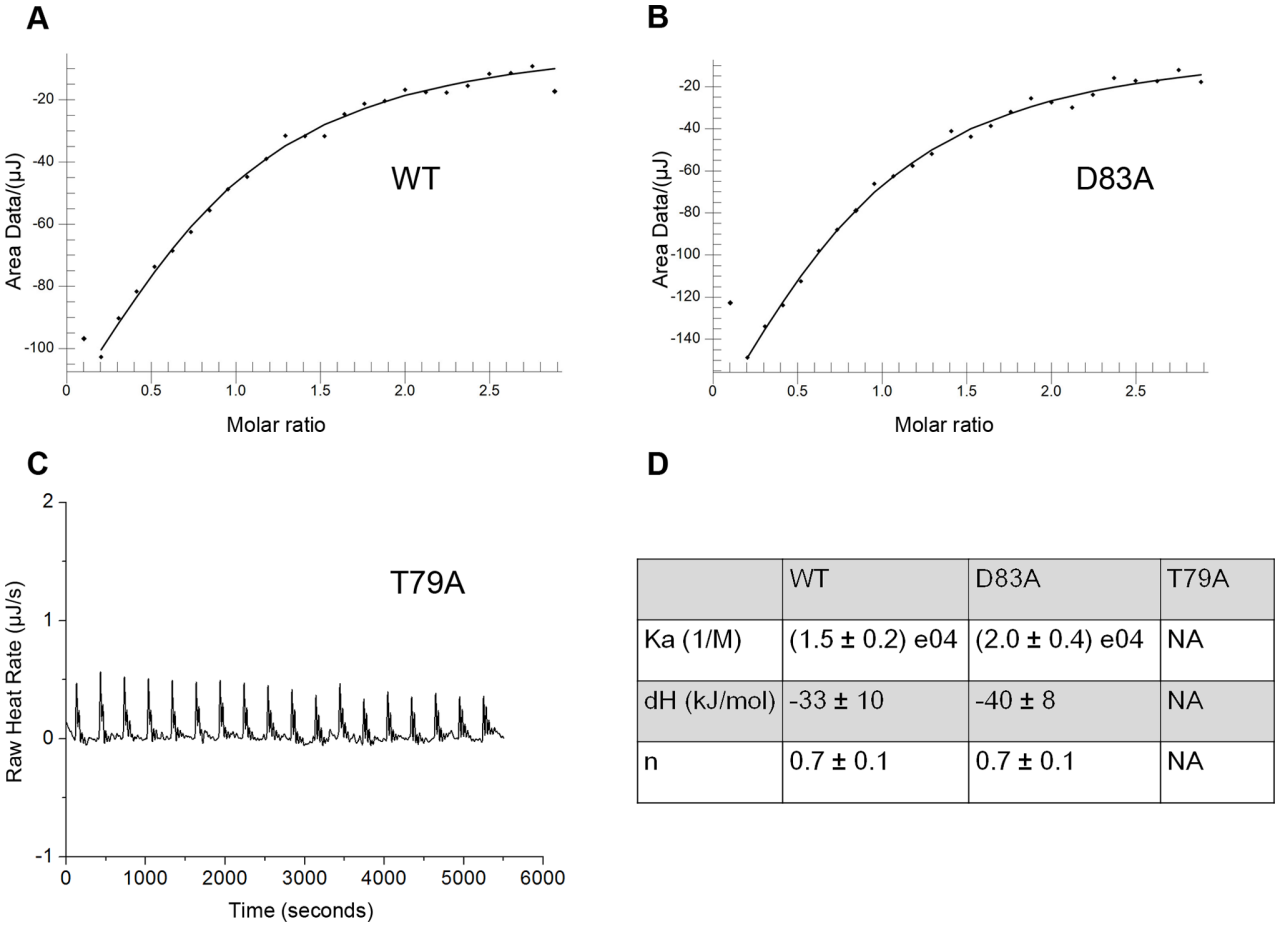
Results from ITC assays of ScRIB7 enzymes. Interactions between enzymes and NADPH were detected by isothermal titration calorimetry using nano-ITC2g. The protein was dialyzed into PBS and diluted to 80 µM. It was then titrated by 800 µM NADPH dissolved in the same buffer (10 µl NADPH was injected every 300 s). The interaction heat flow was monitored to calculate their affinity. Results were analyzed with NanoAnalyze (TA Instruments). A&B, WT and D83A mutant binding curves obtained from plots of the heat from each NADPH injection against injections over time. C, Spikes of heat flow observed when T79A mutant was titrated by NADPH. D, Calculated best fit parameters.

So we generated a series of mutants of ScRIB7 Asp83 and Glu180 and measured their reductase activity. E180A and E180G mutants could not be isolated. Both purified mutants in Glu180 dramatically influenced their catalytic ability (Fig. 6A). D83A carries even much lower activity than E180N or E180Q. Since Asp83 is close to the NADPH binding site, this loss of reduction activity may be caused by reduced NADPH binding ability rather than a deficiency in enzyme catalysis ability. We thus evaluated the affinity of D83A with NADPH with isothermal titration calorimetry (ITC). T79A is a null mutant that probably fails to bind NADPH, so it was used as a negative control. The binding constant of the D83A mutant with NADPH was similar to that of the wild type, while no interaction heat was detected for the T79A mutant (Fig. 7). It thus seems that mutation of Asp83 does not abolish NADPH binding, and the loss of activity in D83A is probably due to failure in other aspects of catalysis. Thus, our data proved ScRIB7 Asp83 and Glu180 are both necessary for the activity of the enzyme.

### Conclusion

Since riboflavin is needed in organisms, microorganisms lacking the riboflavin absorption carrier will dependent on riboflavin biosynthesize pathway. Comparative genomics analysis showed that many bacterial pathogens lack riboflavin absorption pathway [37]. However, analysis over fungi pathogens is also needed to ensure that the riboflavin biosynthesis pathway is their only source of riboflavin. To explore the potential of fungal RBSRs as a potent drug target against pathogens, we have provided the first eukaryotic RBSR structure, and its binary structure with NADPH. Some minor differences in its cofactor binding architecture from bacterial homologs were mentioned. Substrate homologous chemicals with high affinity to dihydrofolate reductases were successfully developed as anti-microbe drugs [10], which brings enlightment in our future research. Further studies are needed to solve complex structure of fungal RBSR with substrate. Activity assays showed that Thr79, Asp83, Glu180 and Gly182 are necessary for the activity of ScRIB7. ScRIB7 catalyzes reduction of DAROPP, while bacterial RibD catalyzes reduction of AROPP. ScRIB7 Thr35 and its corresponding residue, a lysine in bacteria, are important in substrate-specific recognition.

## Supporting Information

Figure S1 Calculated 2Fo-Fc map of cofactor NADPH in ScRIB7 chain A. ScRIB7 chain A is shown as cartoon. NADPH is shown as sticks: carbon (green), oxygen (red), nitrogen (blue). Electron density is carved 1.5 Å around NADPH, and is colored yellow. The 2Fo-Fc map is contoured at 1.5 standard deviation above the mean.
(TIF)

Table S1 Primer sequence. Sequences of primers used in recombinant plasmid preparation and mutagenesis. The subscript F indicates the forward primer, and R indicates the reverse primer. The underlined sequence indicates restriction sites (“GGA TCC” for BamHI, “CTC GAG” for XhoI, and “AGA TCT” for BglII) and mutated sites.
(DOCX)
