# Supplementary figures and images for: Structural and Functional Insights into Saccharomyces cerevisiae Riboflavin Biosynthesis Reductase RIB7
Source: PLoS One. 2013 Apr 19;8(4):e61249. doi: 10.1371/journal.pone.0061249 (PMC3631187; doi:10.1371/journal.pone.0061249)

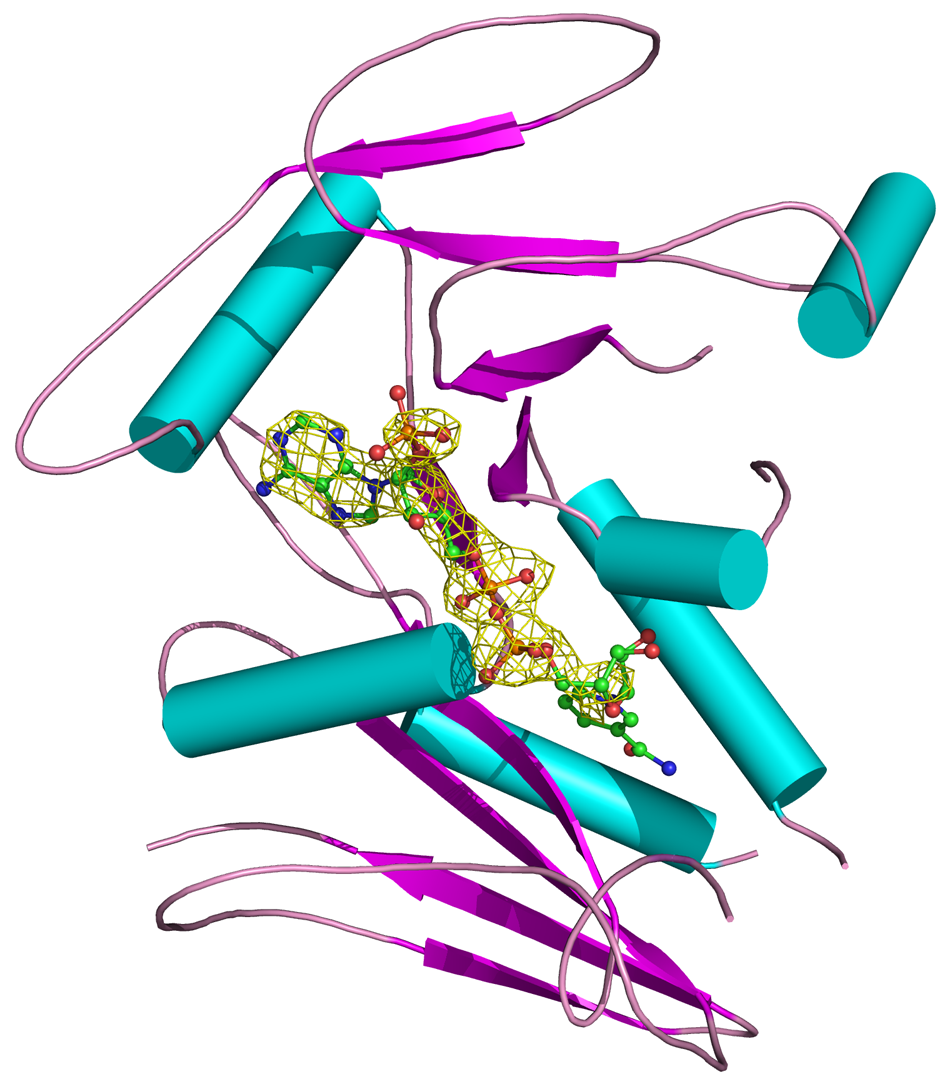

Supplement: Figure S1 — Calculated 2Fo-Fc map of cofactor NADPH in ScRIB7 chain A. ScRIB7 chain A is shown as cartoon. NADPH is shown as sticks: carbon (green), oxygen (red), nitrogen (blue). Electron density is carved 1.5 Å around NADPH, and is colored yellow. The 2Fo-Fc map is contoured at 1.5 standard deviation above the mean. (TIF) [file pone.0061249.s001.tif]
