# Supplementary material for: Structural and Functional Insights into Saccharomyces cerevisiae Riboflavin Biosynthesis Reductase RIB7
Source: PLoS One. 2013 Apr 19;8(4):e61249. doi: 10.1371/journal.pone.0061249 (PMC3631187; doi:10.1371/journal.pone.0061249)
Supplement: Table S1 — Primer sequence. Sequences of primers used in recombinant plasmid preparation and mutagenesis. The subscript F indicates the forward primer, and R indicates the reverse primer. The underlined sequence indicates restriction sites (“GGA TCC” for BamHI, “CTC GAG” for XhoI, and “AGA TCT” for BglII) and mutated sites. (DOCX) [file pone.0061249.s002.docx]

**Table S1:**

Primer information:

| **Primer** | **Sequence** |
| --- | --- |
| EcRibD_F_*: | 5′-CAA GGA TCC ATG CAG GAC GAG TAT TAC ATG-3′, |
| EcRibD_R_: | 5′-CAA CTC GAG TCA TGC ACC CAC TAA ATG C-3′, |
| ScRIB1_F_: | 5′-GGG AGA TCT ATG ACC ATA GAT AAC TAC G-3′, |
| ScRIB1_R_: | 5′-GGG CTC GAG TTA TAT TGC CAG CGT CGA TG-3′, |
| ScRIB7_F_: | 5′-CCC GGA TCC ATG TCT TTG ACA CCA CTG TG-3′, |
| ScRIB7_R_: | 5′-CCC CTC GAG TTA GTC ATC GGC CAG TCT CG-3′, |
| D83A_F_: | 5′-GAA CAG TGC TAG CTG CGA ATC CTG GAT TG-3′ |
| D83A_R_: | 5′-CAA TCC AGG ATT CGC AGC TAG CAC TGT TC-3′ |
| T79A_F_: | 5′-GTA GGA AGT GGA GCG GTG CTA GCT GAT AAT CC-3′ |
| T79A_R_: | 5′-GGA TTA TCA GCT AGC ACC GCT CCA CTT CCT AC-3′ |
| G182T_F_: | 5′-GGT TGA AGG AAC CGC CAA TGT AAT AAA TCA G-3′ |
| G182T_R_: | 5′-CTG ATT TAT TAC ATT GGC GGT TCC TTC AAC C-3′ |
| E180A_F_: | 5′-GTC AGT AAT GGT GCG CGG AGG TGC CAA TG-3′ |
| E180A_R_: | 5′-CAT TGG CAC CTC CGC GCA CCA TTA CTG AC-3′ |
| E180G_F_: | 5′-GTC AGT AAT GGT TGG CGG AGG TGC CAA TG-3′ |
| E180G_R_: | 5′-CAT TGG CAC CTC CGC CAA CCA TTA CTG AC-3′ |
| E180N_F_: | 5′-GTC AGT AAT GGT TAA CGG AGG TGC CAA TG-3′ |
| E180N_R_: | 5′-CAT TGG CAC CTC CGT TAA CCA TTA CTG AC-3′ |
| E180Q_F_: | 5′-GTC AGT AAT GGT TCA GGG AGG TGC CAA TG-3′ |
| E180Q_R_: | 5′-CAT TGG CAC CTC CCT GAA CCA TTA CTG AC-3′ |
| T35K_F_: | 5′-CCT TTG TCA CAC TAA AAT ATG CTC AAT CGC TC-3′ |
| T35K_R_: | 5′-GAG CGA TTG AGC ATA TTT TAG TGT GAC AAA GG-3′ |

*The subscript F indicates the forward primer, and R indicates the reverse primer. The underlined sequence indicates restriction sites (“GGA TCC” for *BamHI*, “CTC GAG” for *XhoI*, and “AGA TCT” for *BglII*) and mutated sites.
